# Accelerated Brain Atrophy in Epilepsy: A Prospective Population‐Based Study

**DOI:** 10.1002/ana.78319

**Published:** 2026-08-24

**Authors:** Marian Galovic, Rebecca S. N. Liu, Alex D. Everitt, Robert Terziev, Louis Lemieux, Matthias J. Koepp, John S. Duncan

**Affiliations:** ^1^ Research Department of Epilepsy, UCL Queen Square Institute of Neurology University College London London UK; ^2^ Department of Neurology, Clinical Neuroscience Center University Hospital and University of Zurich Zurich Switzerland; ^3^ Royal Free London NHS Foundation Trust London UK; ^4^ St. Mary's Hospital, Imperial College Healthcare NHS Trust London UK; ^5^ Department of Neurology Charité – Universitätsmedizin Berlin Berlin Germany

## Abstract

**Objective:**

Previous longitudinal neuroimaging studies suggest that epilepsy is a progressive disorder. To date, these findings have relied largely on populations from tertiary centers, resulting in ascertainment bias, as severely affected individuals are more likely to be rescanned. We aimed to determine rates of progressive brain atrophy in a prospective, population‐based cohort.

**Methods:**

We analyzed longitudinal magnetic resonance imaging (MRI) data from 116 individuals with epilepsy and 89 age‐ and sex‐matched healthy controls recruited from a prospective, longitudinal, community‐based cohort in the United Kingdom. All participants underwent 2 scans 3.5 years apart, regardless of seizure status. We quantified progressive changes in cortical thickness and subcortical volumes using state‐of‐the‐art longitudinal morphometry.

**Results:**

People with epilepsy exhibited accelerated but modest global grey matter volume (GMV) loss compared with controls (2.7 vs 2.4 ml/year; *p* = 0.01). This acceleration was associated with specific phenotypes: focal epilepsy, MRI‐identifiable lesions, and ongoing seizures. In contrast, generalized epilepsy and seizure‐free periods were associated with more stable global trajectories. Cortical regions identified as vulnerable were characterized by markedly accelerated thinning compared to controls (5.3 vs 3.4 μm/year; *p* < 0.001) and were pervasive across most epilepsy phenotypes.

**Interpretation:**

Epilepsy is associated with progressive structural brain damage that exceeds normal aging, but the trajectory is heterogeneous. These prospective population‐based data confirm that vulnerable regions show progressive atrophy, particularly in focal and lesional epilepsy and individuals with ongoing seizures. ANN NEUROL 2026;100:1028–1038

For over a century, patients and their families have asked whether epilepsy causes progressive brain damage. Despite substantial advances in the field, the answer remains controversial.^1^, ^2^ Increasing evidence from cross‐sectional and longitudinal neuroimaging challenges the traditional view of epilepsy as a static disorder, suggesting that it is a dynamic process associated with progressive structural alterations.^3^ In tertiary referral cohorts, we and others have demonstrated that focal epilepsy is associated with accelerated cortical thinning and hippocampal atrophy that outpaces the trajectory of normal aging.^3^, ^4^, ^5^, ^6^, ^7^, ^8^ These structural changes appear dynamic, and potentially modifiable by successful surgical intervention.^8^

A critical limitation, however, undermines the generalizability of these findings: ascertainment bias. Existing longitudinal studies have almost exclusively focused on individuals attending specialized epilepsy centers. In these clinical cohorts, repeat neuroimaging is not performed randomly but is triggered by clinical necessity, typically due to drug resistance, worsening seizure control, cognitive impairment, or presurgical evaluation. Consequently, current literature is skewed toward the most severe end of the spectrum of the disease, whereas those with well‐controlled epilepsy or benign courses are under‐represented because they rarely require follow‐up imaging. Thus, it remains unknown whether progressive brain atrophy is an intrinsic feature of all epilepsies or a specific consequence of severe, refractory disease.

To discern the true natural history of structural changes in the general epilepsy population, we revisited a unique, prospective, population‐based cohort.^9^, ^10^, ^11^, ^12^ Unlike tertiary cohorts, this study systematically rescanned community‐based patients regardless of their clinical status, thereby circumventing selection bias. The original analyses of this dataset relied on volumetric methods available in the early 2000s, over 2 decades ago.^11^, ^12^ Here, we applied state‐of‐the‐art longitudinal morphometric processing to these prospective population‐based data to provide more generalizable estimates of progressive structural changes in epilepsy, determining whether accelerated atrophy is a generalizable phenomenon or limited to specific clinical phenotypes.

## Methods

### Study Population

We analyzed data from a prospective, community‐based longitudinal study designed to assess cerebral structural changes in a representative epilepsy population.^9^, ^10^, ^11^, ^12^ The sampling frame comprised all individuals registered with participating primary care practices, and the manual and electronic audit was designed to capture all cases from this community base rather than from tertiary referral pathways. Participants were recruited prospectively over a 2‐year period between June 1995 and May 1997 from a local population of 207,553 individuals under regular active surveillance by 21 general medical practices in the United Kingdom. The study included individuals aged 15 years or older with a confirmed diagnosis of established epilepsy. Case identification was optimized through an intensive manual and electronic audit of primary care records, screening for new antiseizure medication prescriptions or epilepsy‐related diagnostic codes. For subgroup analyses, the cohort was stratified by epilepsy syndrome (focal vs generalized), magnetic resonance imaging (MRI) findings (lesional vs nonlesional), and seizure status during the follow‐up period (seizure‐free vs ongoing seizures).

The control population consisted of age‐ and sex‐matched healthy controls, comprising volunteers, hospital staff, and friends or spouses of patients, recruited from the same community base.

Exclusion criteria included isolated seizures without a definitive diagnosis of epilepsy, acute symptomatic seizures only, progressive neurological disorders, significant psychiatric comorbidity, alcohol or drug abuse, MRI contraindications, MRI of poor quality (eg, movement artifacts), and failed magnetic resonance (MR) postprocessing.

All participants underwent baseline neuroimaging and were invited for a repeat scan after a target interval of 3.5 years. Patient recall was optimized through an active monitoring system involving 6‐month reviews of general practice and outpatient records. Crucially, the follow‐up scan was performed regardless of seizure outcome or clinical course, ensuring that patients without seizure remission were as likely to be rescanned as those with drug‐resistant epilepsy.

Attrition rate in the original follow‐up study was 20%.^9^, ^10^, ^11^, ^12^ Reasons for attrition were death (20%), infirmity (9%), inability to trace (13%), emigration (7%), pregnancy (2%), inability to tolerate scanning (6%), and refusal (43%).

Among 184 participants with 2 scans in the original follow‐up study, we excluded 21 with isolated unprovoked seizures without confirmed epilepsy, 11 with acute symptomatic seizures only, and 3 with unknown epilepsy classification, leaving 149 individuals with confirmed focal or generalized epilepsy. Imaging quality control then excluded 33 individuals: 18 because of large lesions (>30 ml by ABC/2 formula) likely to disrupt preprocessing, 1 because of a surgical resection cavity, 1 because of MRI artifact, 12 because of severe movement artifact, and 1 because of failed quality control (see below) with poor segmentation on visual review. No controls with 2 scans were excluded.

### Standard Protocol Approvals, Registrations, and Patient Consents

The study protocol was approved by the Joint Ethics Committee of the National Hospital for Neurology and Neurosurgery and the Institute of Neurology, London, United Kingdom. Written informed consent was obtained from all participants prior to inclusion.

### MRI Data Acquisition

Longitudinal neuroimaging data were acquired on a 1.5T General Electric Signa Horizon scanner (GE Medical Systems, Milwaukee, WI) at the Chalfont Centre for Epilepsy, Chalfont St. Peter, UK. To prevent scanner‐induced variance, baseline and follow‐up scans were performed on the same scanner without hardware or software upgrades using identical acquisition sequences. T1‐weighted structural images were acquired using a fast inversion recovery prepared 3D spoiled gradient echo (IR‐SPGR) volume sequence (TR/TE/TI/NEX = 17.4/4.2/450/1; flip angle = 20 degrees; matrix = 256 × 192; and field of view [FOV] = 24 × 18 cm). The acquisition consisted of 124 contiguous coronal 1.5‐mm‐thick slices, providing whole‐brain coverage for detailed morphometric analysis.

### Image Processing and Longitudinal Morphometry

We estimated total intracranial volume, grey and white matter volumes, cortical thickness, and regional subcortical volumes for all scans in all cohorts using the fully automated,^13^ validated,^13^, ^14^, ^15^ and reliable^13^, ^15^ Computational Anatomy Toolbox (CAT12; Jena University Hospital, Germany; https://neuro-jena.github.io/cat/) within Statistical Parametric Mapping 12 (SPM12; Wellcome Centre for Human Neuroimaging, United Kingdom; https://www.fil.ion.ucl.ac.uk/spm/software/spm12/) running in MATLAB R2023b (The MathWorks, Natick, MA) with an inverse‐consistent longitudinal registration approach.^16^ The CAT12 toolbox previously showed excellent test–retest reliability (*R* ^2^ = 0.986) and was validated against other cortical surface reconstruction methods, showing fewer thickness measurement errors than comparable approaches.^14^, ^15^

Cortical thickness was computed using CAT12's projection‐based thickness method using standard settings, which derives white matter distance from tissue segmentation obtained using geodesic shooting and projects local maxima of thickness to grey matter voxels, effectively accounting for partial volume effects and sulcal geometry. The pipeline comprises topology correction using spherical harmonics, spherical mapping to a common coordinate system, diffeomorphic surface registration for inter‐subject alignment, and subsequent smoothing with a 12 mm surface‐based kernel.

Subcortical regional volumes in 5 regions (hippocampus, amygdala, thalamus, pallidum, and putamen) were calculated by projecting grey matter volume (GMV) segmentations onto the Hammers brain atlas in template space.^17^

All data underwent CAT12‐based quality control, and scans with signs of excessive misalignment, misregistration, inaccurate estimations, or large lesions that may disrupt processing were excluded as detailed above. Image quality was assessed by evaluating image noise, inhomogeneities, and resolution within the CAT12 quality assurance framework.

### Statistical Analysis

We estimated the rate of GMV, cortical thickness, and subcortical volume change using linear mixed‐effects models (Fig 1), a flexible framework for longitudinal analysis of multiple repeated measurements per subject with irregular measurement intervals. Consistent with neuroimaging convention, we operationalized reductions in GMV and cortical thickness as “atrophy,” acknowledging that in the absence of histological validation, these macroscopic volume losses may reflect reduction of neuropil rather than neuronal loss, per se.^18^ Vulnerable cortical regions were defined as those areas that showed significantly accelerated cortical thinning in epilepsy compared with healthy controls (Fig 2).

**FIGURE 1:**
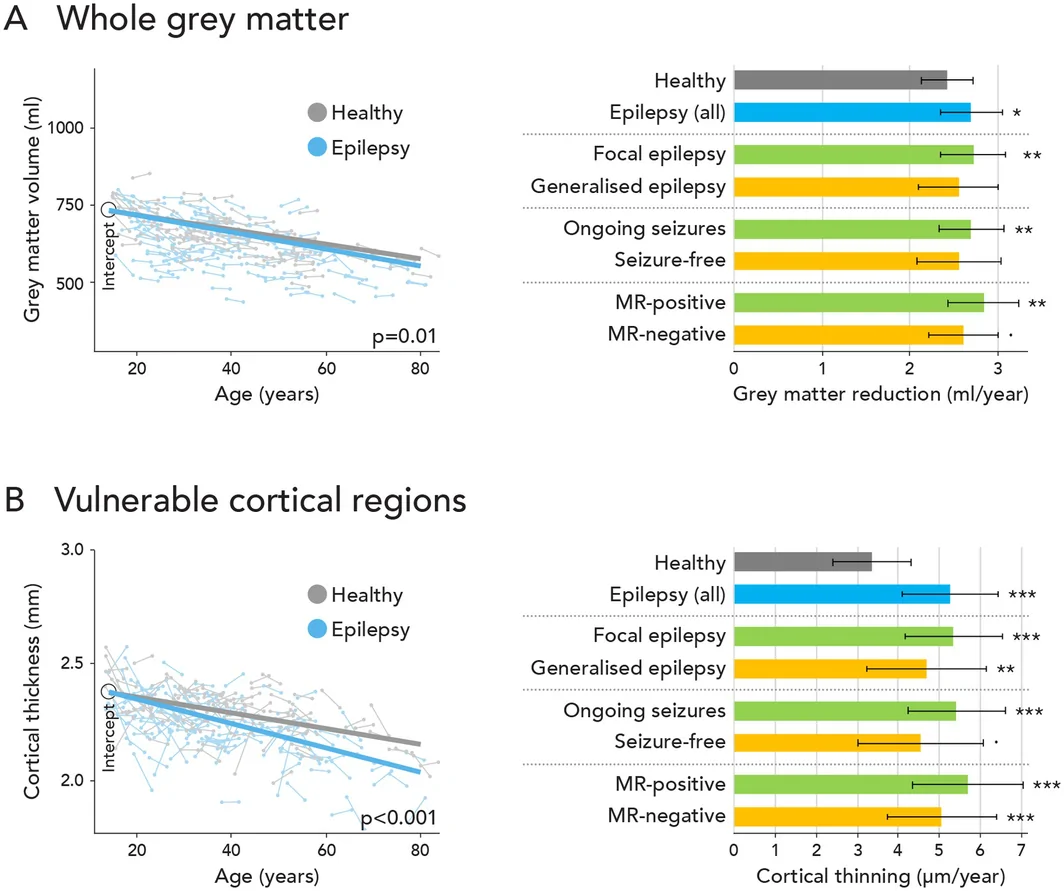
Accelerated global and regional atrophy in epilepsy. (A) Global grey matter volume changes over time. Scatterplots show individual pairs of data points and linear regression lines for healthy controls and people with epilepsy, demonstrating a significantly steeper rate of volume loss in epilepsy (*p* = 0.01). Bar charts display the estimated annual rate of grey matter loss (ml/year) adjusted for covariates, stratified by clinical phenotype. (B) Progressive cortical thinning in “vulnerable cortical regions” (areas showing significant group differences). Scatterplots and bar charts illustrate markedly accelerated thinning in epilepsy compared with controls (*p* < 0.001), which was found across most subgroups. Error bars represent standard errors. Asterisks indicate significant acceleration compared to healthy controls: ° *p* < 0.10, * *p* < 0.05, ** *p* < 0.01, *** *p* < 0.001. [Color figure can be viewed at www.annalsofneurology.org]

**FIGURE 2:**
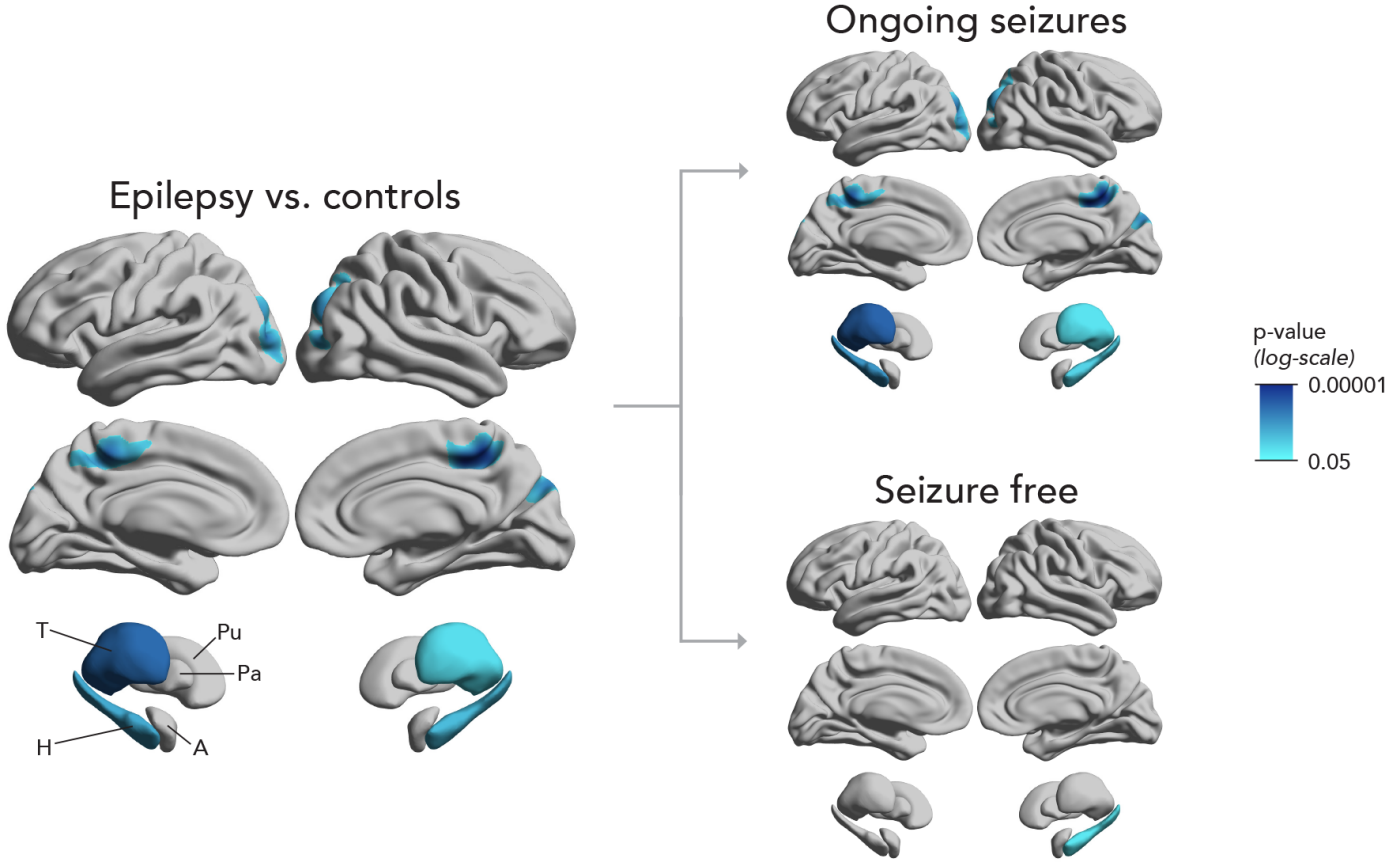
Spatial topography of accelerated cortical thinning and subcortical volume loss. Brain surface maps and subcortical renderings display cortical and subcortical areas with significantly accelerated atrophy in people with epilepsy compared to age‐ and sex‐matched healthy controls. The significance bar indicates the statistical significance (log‐scaled *p* values, family wise error corrected), indicating accelerated atrophy. Results are shown for the whole epilepsy cohort (left), individuals with ongoing seizures (right top), and seizure‐free individuals (right bottom). A = amygdala; H = hippocampus; Pa = pallidum; Pu = putamen; T = thalamus. [Color figure can be viewed at www.annalsofneurology.org]

For each non‐overlapping comparison, we fitted a separate model with the same architecture. The primary term of interest was the interaction between the variable of interest (ie, group allocation or clinical variable) and age at MRI scan; the corresponding *p* values therefore test whether longitudinal slopes differed between groups or across clinical variables. All models included a random effect of participant, fixed effects of age at scan, sex, and group, and the relevant interaction with age at scan. With this approach, we were able to test for within‐participant morphometric changes over time while correcting for baseline demographic differences and for different interscan intervals. Volumetric data (GMV and subcortical volumes) were additionally corrected by including total intracranial volume as a covariate. Models comparing clinical variables within participants with epilepsy were additionally corrected for seizure frequency in between MRI scans, history of tonic–clonic seizures, presence of hippocampal sclerosis, duration of epilepsy at baseline, and number of antiseizure medications at baseline, except when the covariate itself was the variable of interest.

We report findings considered significant at *p* < 0.05 corrected for multiple comparisons using random field theory for nonisotropic images on a cluster level^19^ for cortical thickness and after Bonferroni correction for the 10 subcortical volumes tested (corrected *p*‐values shown in manuscript).

We performed statistical analyses using R software version 4.4.1 (R Foundation for Statistical Computing, Vienna, Austria). Cortical thickness and subcortical volume calculations were performed using SurfStat (https://www.math.mcgill.ca/keith/surfstat/) in MATLAB.

## Results

### Study Population

The final analysis comprised 116 individuals with confirmed epilepsy (median age at baseline scan = 34 years, interquartile range = 25–49; 63 female subjects) and 89 healthy controls (median age = 35 years, interquartile range = 27–48; 40 female subjects; see the Table 1). Within the epilepsy cohort, 85 individuals (73%) were classified as having focal epilepsy, whereas 31 (27%) had generalized epilepsy. Structural MRI abnormalities, including hippocampal sclerosis, were identified in 40 participants (34%). During the follow‐up interval, 90 individuals (78%) continued to experience seizures, whereas 26 (22%) remained seizure‐free. Among individuals with focal epilepsy, seizure focus laterality was left‐sided in 16, right‐sided in 8, bilateral in 13, and unknown or undetermined in 48.

**Table 1:** Demographic and clinical characteristics

	Focal Epilepsy, n = 85	Generalized Epilepsy, n = 31	Healthy Controls, n = 89
Sex			
M	42 (49%)	11 (35%)	49 (55%)
F	43 (51%)	20 (65%)	40 (45%)
Age and duration, yr			
Age at baseline scan	37 (27–50)	28 (23–37)	35 (27–48)
Age at first afebrile seizure	20 (10–30)	10 (6–15)	
Duration of epilepsy	15 (6–25)	16 (11–27)	
Imaging			
Lesional, incl. HS	36 (42%)	4 (13%)	
HS	17 (20%)	0 (0%)	
Epilepsy localization			
Temporal lobe	51 (60%)	0 (0%)	
Frontal lobe	8 (9%)	0 (0%)	
Generalized	0 (0%)	31 (100%)	
Unknown/undetermined	26 (31%)	0 (0%)	
Seizures			
Seizure frequency, any type, per yr	27 (0–150)	92 (6–338)	
Seizure frequency, any seizures, categoric			
No seizures	22 (27%)	4 (13%)	
Less than once a mo	17 (21%)	5 (17%)	
Once a mo to once a wk	16 (20%)	6 (20%)	
Once a wk to daily	26 (32%)	15 (50%)	
Daily seizures	0 (0%)	0 (0%)	
Tonic–clonic seizures			
History of tonic–clonic seizures	52/81 (64%)	25/30 (83%)	
Tonic–clonic seizures in last 6 mo	34/81 (42%)	21/30 (70%)	
Tonic–clonic seizure frequency, per yr	0 (0–5)	3 (0–16)	
No. of ASMs at baseline	1 (1–2)	1 (1–2)	

Data are presented as n (%) or median (interquartile range). Data on tonic–clonic seizures were available for 81 individuals with focal epilepsy and 30 individuals with generalized epilepsy. ASMs = antiseizure medications; HS = hippocampal sclerosis; incl. = including.

### Global and Regional Rates of Atrophy

Analysis of global GMV reduction demonstrated a significantly faster rate of loss in people with epilepsy (2.7 ± 0.2 ml/year; *p* = 0.01 for the epilepsy‐by‐age‐at‐scan interaction) compared with healthy controls (2.4 ± 0.2 ml/year; see Fig 1A); rate difference = 0.27 ml/year (95% confidence interval = 0.06–0.49). Subgroup stratification indicated that this accelerated GMV loss compared with healthy aging was driven by specific phenotypes: it was significant in focal epilepsy (2.7 ± 0.2 ml/year; *p* = 0.005) but not generalized epilepsy (2.5 ± 0.2 ml/year; *p* = 0.37); in those with ongoing seizures (2.7 ± 0.2 ml/year; *p* = 0.01) but not seizure‐free individuals (2.6 ± 0.2 ml/year; *p* = 0.23); and in MRI‐positive (2.8 ± 0.2 ml/year; *p* = 0.004) but not MRI‐negative individuals (2.6 ± 0.2 ml/year; *p* = 0.08). There were no differences in atrophy rates between male and female patients with epilepsy.

Given the heterogeneous distribution of epilepsy‐related structural changes, we further quantified atrophy rates within “vulnerable cortical regions,” areas defined by the vertex‐wise analysis as showing significantly accelerated thinning in the overall epilepsy group (see Fig 2); this analysis was used to summarize the magnitude of change in affected regions rather than as an independent topographic test. In these regions, the rate of cortical thinning was markedly greater in people with epilepsy (5.3 ± 0.6 μm/year; *p* < 0.001) relative to healthy controls (3.4 ± 0.5 μm/year; see Fig 1B); rate difference = 1.9 μm/year (95% confidence interval = 1.2–2.5). Unlike global GMV, accelerated thinning in vulnerable regions was more pervasive across subtypes: it was significant in both focal (5.3 ± 0.6 μm/year; *p* < 0.001) and generalized epilepsies (4.7 ± 0.7 μm/year; *p* = 0.005), and in both MRI‐positive (5.7 ± 0.7 μm/year; *p* < 0.001) and MRI‐negative cases (5.0 ± 0.7 μm/year; *p* < 0.001). Among those with ongoing seizures, thinning was accelerated (5.4 ± 0.6 μm/year; *p* < 0.001), whereas seizure‐free individuals showed a non‐significant trend toward acceleration (4.5 ± 0.8 μm/year; *p* = 0.07). Among the 26 seizure‐free individuals, 22 had focal epilepsy and 4 had generalized epilepsy.

### Topography of Progressive Damage

Vertex‐wise longitudinal morphometry revealed specific patterns of accelerated atrophy in people with epilepsy compared with healthy controls (see Fig 2). Significant clusters were identified in the right (1,021 vertices; *p* < 0.001) and left (605 vertices; *p* = 0.02) parieto‐occipital junctions, as well as the right (557 vertices; *p* = 0.004) and left (509 vertices; *p* = 0.008) medial pericentral cortices. Subcortical analysis demonstrated significant progressive volume loss in the left (*t* = 3.7; *p* < 0.001) and right (*t* = 2.2; *p* = 0.01) thalami, and the left (*t* = 3.0; *p* = 0.001) and right (*t* = 2.7; *p* = 0.003) hippocampi. No areas of progressive hypertrophy^20^ were detected in the epilepsy group compared with healthy aging in controls.

When stratified by seizure status, individuals with ongoing seizures (n = 90) exhibited a widespread topographic pattern similar to the overall cohort. In contrast, seizure‐free individuals (n = 26) displayed a restricted phenotype, with accelerated atrophy confined to the right hippocampus (*t* = 2.1; *p* = 0.02). The laterality of epilepsy also influenced the spatial distribution of damage (Fig 3). People with left‐lateralized epilepsy showed accelerated atrophy restricted to the left hemisphere, whereas those with right‐lateralized epilepsy exhibited accelerated atrophy of the right hippocampus and bilateral thalami, with a right‐sided predominance.

**FIGURE 3:**
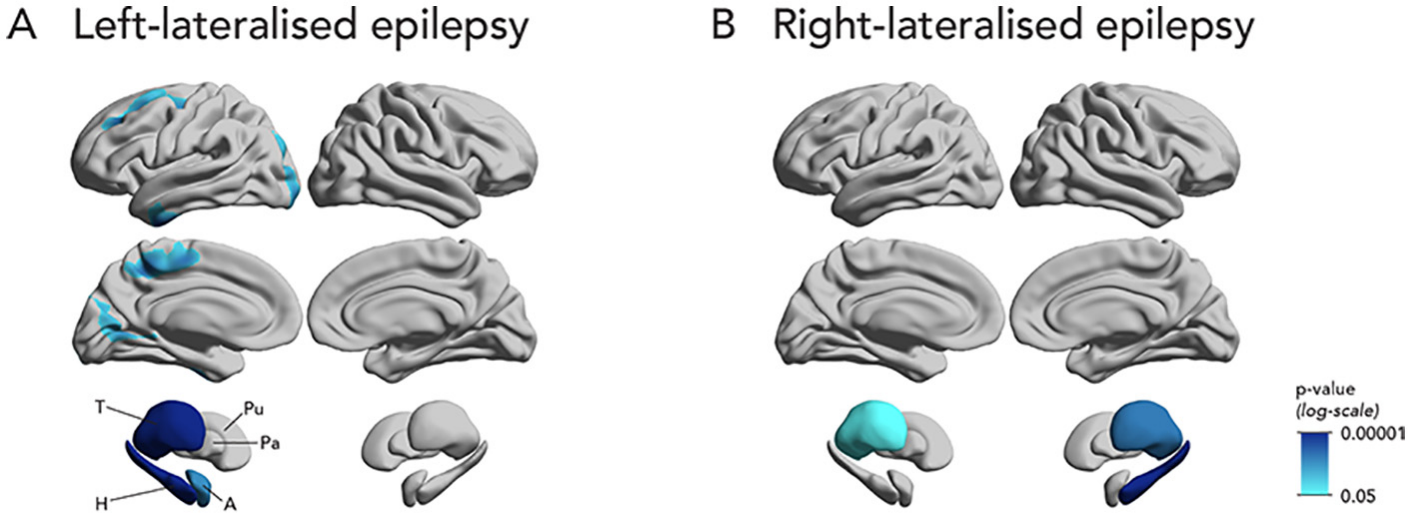
Effect of epilepsy laterality on atrophy patterns. Brain surface maps and subcortical renderings display cortical and subcortical areas with significantly accelerated atrophy in patients with left‐lateralized epilepsy (A) and right‐lateralized epilepsy (B) compared with healthy controls. The significance bar indicates the statistical significance (log‐scaled *p* values, family wise error corrected), indicating accelerated atrophy. A = amygdala; H = hippocampus; Pa = pallidum; Pu = putamen; T = thalamus. [Color figure can be viewed at www.annalsofneurology.org]

Regarding epilepsy syndrome (Fig 4), no significant clusters of accelerated atrophy were detected in the generalized epilepsy group (n = 31). Conversely, people with focal epilepsy (n = 85) displayed the characteristic pattern observed in the overall group, with additional involvement of the superior frontal gyrus. Within focal epilepsy, MRI‐positive individuals (n = 36) showed extensive changes similar to the overall focal cohort, whereas MRI‐negative focal epilepsy (n = 49) was associated with a more limited pattern involving only the right medial pericentral cortex and the left thalamus.

**FIGURE 4:**
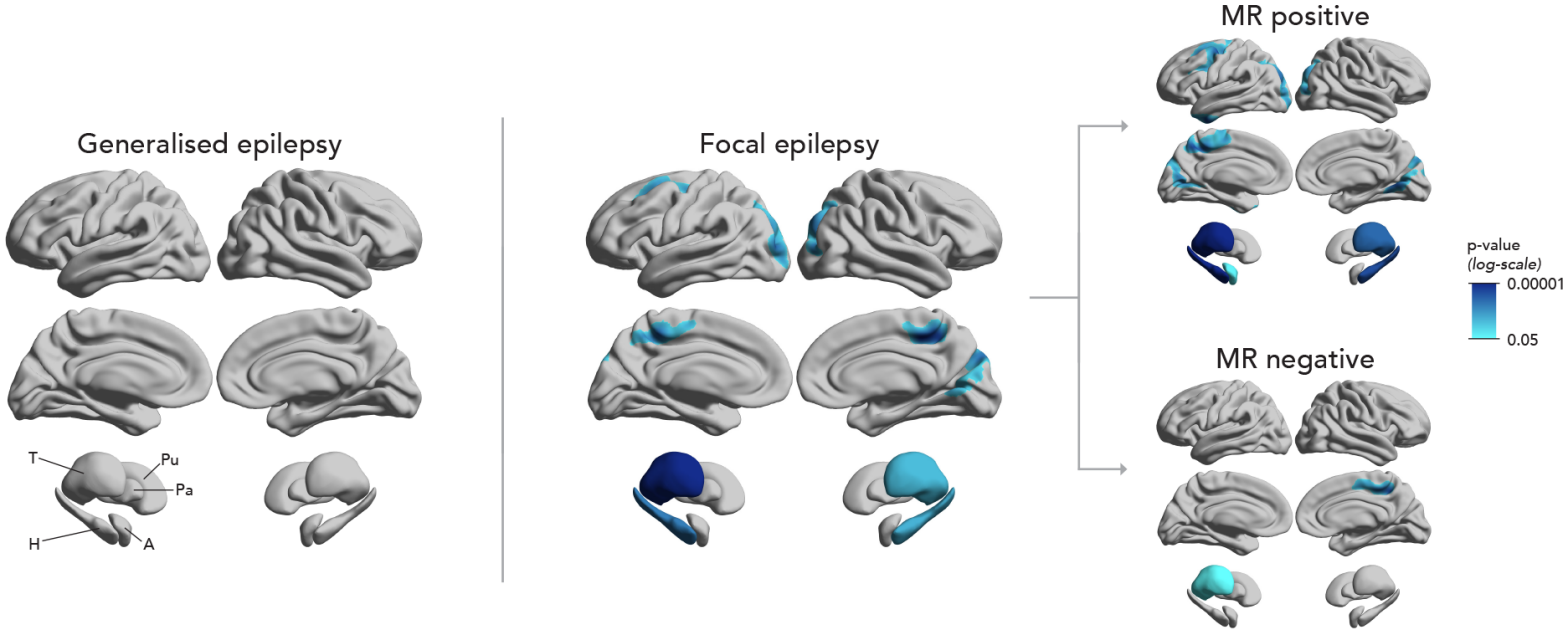
Patterns of progressive atrophy by syndrome and etiology. Brain surface maps and subcortical renderings display cortical and subcortical areas with significantly accelerated atrophy compared with healthy controls, stratified by epilepsy syndrome (generalized vs focal) and MRI status (MR‐positive vs MR‐negative). Accelerated atrophy is prominent in focal and MR‐positive epilepsies but not detectable in generalized epilepsy. The significance bar indicates the statistical significance (log‐scaled *p* values, family wise error corrected), indicating accelerated atrophy. A = amygdala; H = hippocampus; MR = magnetic resonance; MRI = magnetic resonance imaging; Pa = pallidum; Pu = putamen; T = thalamus. [Color figure can be viewed at www.annalsofneurology.org]

### Clinical Determinants of Progressive Atrophy

To delineate the clinical drivers of progressive damage, we performed multivariable linear mixed effects analyses within the cohort of people with epilepsy (n = 116). Higher seizure frequency was associated with accelerated atrophy of the bilateral putamina and a nonsignificant trend toward faster atrophy of the right frontal operculum; conversely, it was associated with slower atrophy of the right hippocampus (Fig 5A). A history of tonic–clonic seizures was predictive of accelerated atrophy in the right hippocampus, thalamus, and amygdala (Fig 5B). The presence of hippocampal sclerosis was associated with more rapid atrophy of the hippocampi and the left amygdala (Fig 5C). Shorter duration of epilepsy was associated with accelerated atrophy of the right putamen and bilateral pallida (Fig 5D), whereas a higher baseline load of antiseizure medications was associated with accelerated atrophy of the right hippocampus and thalamus (Fig 5E).

**FIGURE 5:**
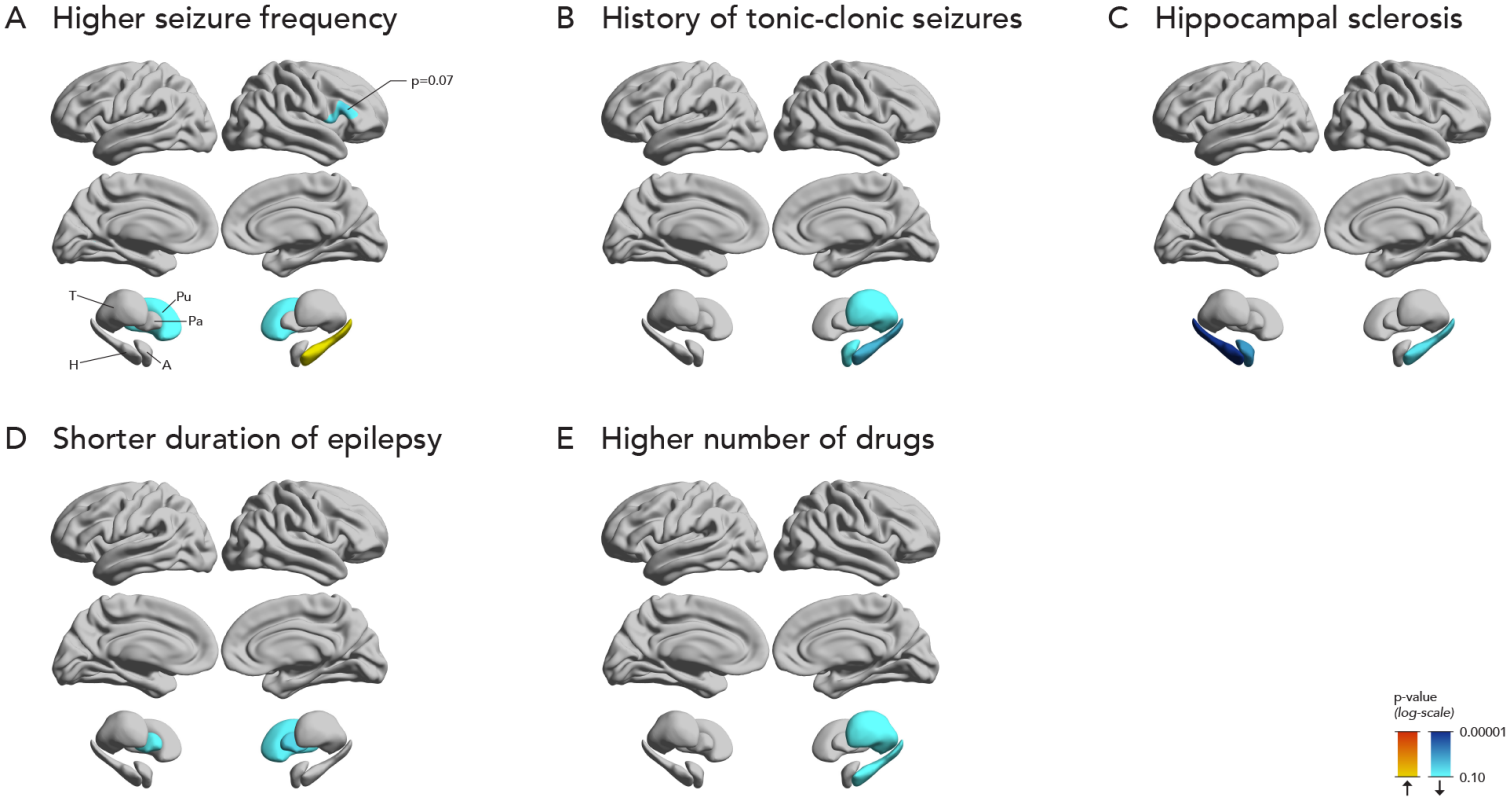
Clinical determinants of progressive atrophy. Brain surface maps and subcortical renderings illustrating the correlation between clinical variables and the rate of atrophy within the epilepsy cohort. (A) Higher seizure frequency is associated with accelerated atrophy of the putamina but slower atrophy of the right hippocampus. (B) History of generalized tonic–clonic seizures predicts accelerated atrophy of the right hippocampus, amygdala, and thalamus. (C) Hippocampal sclerosis is associated with accelerated bilateral hippocampal and left amygdala atrophy. (D) Shorter disease duration is associated with accelerated basal ganglia atrophy. (E) Higher number of antiseizure medications is associated with right thalamic and hippocampal atrophy. The significance bar indicates the statistical significance (log‐scaled *p* values, family wise error corrected), indicating accelerated atrophy or hypertrophy. A = amygdala; H = hippocampus; Pa = pallidum; Pu = putamen; T = thalamus. [Color figure can be viewed at www.annalsofneurology.org]

Direct comparisons between clinical subgroups further clarified these associations (Fig 6). People with focal epilepsy exhibited significantly accelerated atrophy in the bilateral thalami and left hippocampus compared with those with generalized epilepsy (see Fig 6A). Individuals with ongoing seizures showed more rapid atrophy of the bilateral putamina but slower atrophy of the right hippocampus compared with seizure‐free individuals (see Fig 6B). Finally, MRI‐positive individuals demonstrated significantly accelerated atrophy in the bilateral hippocampi, thalami, and amygdalae, along with a nonsignificant trend in the left precentral gyrus, compared with MRI‐negative cases (see Fig 6C).

**FIGURE 6:**
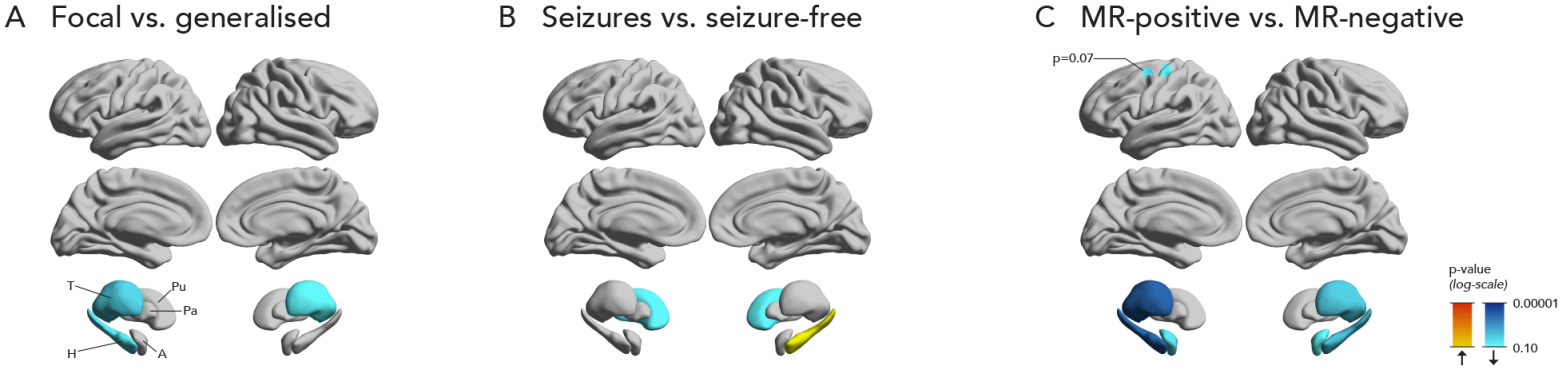
Direct comparisons of atrophy rates between clinical phenotypes. Brain surface maps and subcortical renderings showing areas where atrophy rates significantly differ between subgroups. (A) Focal epilepsy shows faster thalamic and left hippocampal atrophy compared to generalized epilepsy. (B) Ongoing seizures are associated with faster putaminal atrophy but slower right hippocampal atrophy compared to seizure‐free individuals. (C) MR‐positive individuals show faster atrophy of the hippocampi, amygdalae, and thalami compared with MR‐negative individuals. The significance bar indicates the statistical significance (log‐scaled *p* values, family wise error corrected), indicating accelerated atrophy or slower atrophy. A = amygdala; H = hippocampus; MR = magnetic resonance; Pa = pallidum; Pu = putamen; T = thalamus. [Color figure can be viewed at www.annalsofneurology.org]

## Discussion

We provide more generalizable, state‐of‐the‐art morphometric estimates of progressive structural changes in epilepsy by re‐analyzing a unique, prospective, population‐based cohort. Our findings confirm that epilepsy is associated with accelerated brain atrophy compared to normal aging, and also highlight the crucial influence of clinical phenotype. Progressive atrophy was not uniform; it was significantly more pronounced in individuals with focal epilepsy, ongoing seizures, and MRI‐identifiable lesions. Conversely, people with generalized epilepsy, those who were seizure‐free, and MRI‐negative cases exhibited lower rates of global atrophy, although they still demonstrated subtle, accelerated thinning in spatially restricted vulnerable cortical regions. By avoiding the ascertainment bias inherent to tertiary referral cohorts, these data offer a more realistic and generalizable view of the natural history of epilepsy, suggesting that although progressive damage is a core feature of the disorder, its severity is highly heterogeneous and driven by disease activity and etiology.

A primary contribution of this study is the quantification of atrophy rates in a prospective population‐based cohort. We observed accelerated whole‐brain grey matter loss in epilepsy compared with healthy aging, yet the magnitude of this loss was lower than that reported in previous hospital‐based longitudinal studies.^7^, ^8^ This discrepancy is likely attributable to referral bias in earlier works: studies based in tertiary centers inevitably select for individuals with refractory, severe disease who require repeat neuroimaging, thereby inflating estimates of progression. By prospectively rescanning a community‐based cohort regardless of clinical status, we demonstrate that the “average” rate of progression in the broader epilepsy population is more modest. However, when we restricted our analysis to “vulnerable cortical regions,” specific areas like the parieto‐occipital and medial pericentral cortices, the rates of thinning approached those observed in tertiary cohorts.^7^, ^8^ This suggests that epilepsy‐related atrophy is topographically specific; whereas global brain health may be relatively preserved in the wider population, specific networks remain uniquely vulnerable to progressive damage across the spectrum of disease severity.

The trajectory of structural decline was strongly influenced by the specific epilepsy syndrome. Progressive global atrophy was prominent in focal epilepsy, particularly in lesional cases, but was attenuated in generalized epilepsy. This divergence supports the hypothesis that the underlying topology of the epileptic network drives structural progression.^21^ Cross‐sectional studies from the ENIGMA consortium have similarly reported less severe widespread atrophy in generalized compared with focal epilepsies.^22^ Our longitudinal data extend this by showing that generalized epilepsy is associated with a more stable structural trajectory over time, despite these individuals often experiencing a higher frequency of seizures, including generalized tonic–clonic seizures. We acknowledge that our generalized epilepsy cohort (n = 31) was smaller than the focal group, which may limit statistical power. The generalized cohort was also younger compared to the focal group, and an older brain may be more vulnerable to atrophy.^7^ However, direct statistical comparisons corrected for age revealed significantly greater subregional atrophy in focal epilepsy, suggesting this dissociation reflects true biological differences rather than statistical power alone. This implies that the anatomic substrate and etiology of the epilepsy are perhaps more critical determinants of progressive damage than seizure burden alone.

The relationship between seizure frequency and structural progression remains a subject of intense debate, with conflicting evidence supporting^3^, ^23^ or refuting^5^, ^7^, ^11^ a direct dose‐dependent effect. In our cohort, individuals with ongoing seizures exhibited more rapid global and subregional atrophy than those who were seizure‐free. Furthermore, higher seizure frequency was associated with accelerated atrophy in the putamina. However, interpreting this association is complex. It remains unclear whether seizures directly cause excitotoxic damage or whether a high seizure frequency is merely a marker of a more severe, intrinsically progressive form of disease. Conversely, the fact that we also detected trends toward accelerated atrophy in seizure‐free individuals suggests that mechanisms independent of overt seizures, such as subclinical epileptiform activity, effects of interictal discharges,^24^ underlying network dysfunction,^21^ or chronic antiseizure medication use, may also contribute to progressive neurodegeneration.^25^, ^26^

Thus, it remains unclear whether the accelerated atrophy observed in active epilepsy is a direct consequence of seizures or a marker of intrinsic disease severity. Resolving this would require longitudinal intra‐individual monitoring across periods of seizure freedom and ongoing seizures, a design not feasible in the current study. Interestingly, we observed a complex relationship in the hippocampus. Although global atrophy was faster in those with seizures, the rate of atrophy in the right hippocampus was paradoxically less in those with continuing seizures.

Our multivariable analyses identified several other critical predictors of progression. A history of generalized tonic–clonic seizures and a higher burden of antiseizure medications were both associated with accelerated subcortical atrophy, likely serving as proxies for disease severity. Consistent with previous literature,^7^, ^27^ the presence of hippocampal sclerosis predicted rapid ongoing atrophy of the hippocampus and amygdala, indicative of an active, progressive lesion. Notably, shorter epilepsy duration at baseline was associated with accelerated atrophy in the basal ganglia. This suggests that morphological changes may be most dynamic during the early stages of the disease, decelerating as the condition becomes chronic. This pattern is consistent with our previous findings in tertiary cohorts^7^ and has profound clinical implications, reinforcing the argument for early intensive therapeutic intervention to arrest structural decline. In this regard, we have previously demonstrated that successful epilepsy surgery normalizes progressive atrophy to the rate of healthy aging,^8^ but it remains unknown whether effective medical treatment offers similar neuroprotective benefits.

Our results refine the original reports from this cohort, which detected progressive neocortical volume loss in chronic epilepsy^11^ but found no significant group‐level changes in newly diagnosed seizures.^10^ Although the original subtraction‐based methods successfully identified gross atrophy, they lacked the spatial resolution to detect the subtle, topographically specific cortical thinning we observed here, particularly the relationship with seizure activity. The application of modern vertex‐wise morphometry has thus unveiled a more complex landscape, demonstrating that structural damage is dynamic and driven by specific phenotypes rather than being a uniform consequence of the diagnosis.

This study has limitations. First, separating the effects of the disease from the effects of treatment is challenging; most individuals with epilepsy were exposed to antiseizure medications, which may additionally influence brain structure.^25^, ^26^ Second, we lacked longitudinal neuropsychological data to determine if these morphometric changes correlate with cognitive decline. Third, with only 2 time points, we could not assess nonlinear trajectories of atrophy or the specific effects of fluctuating seizure control within the same individual. Fourth, imaging was performed on a 1.5T scanner; whereas the strict preservation of hardware was a methodological asset for longitudinal consistency, higher field strengths would likely offer greater sensitivity to subtle pathology. Fifth, the morphometric pipeline could not be applied reliably to individuals with large lesions, surgical cavities, severe movement artifacts, or failed segmentation; excluding such participants was necessary for valid morphometric estimation but may limit claims about the most structurally abnormal cases.

In conclusion, this prospective population‐based study establishes that epilepsy is a progressive disorder of the brain, characterized by accelerated structural atrophy that extends beyond the limits of normal aging. However, we demonstrate that this progression is not a uniform fate but a highly variable trajectory driven by syndrome, etiology, and disease activity. By reducing referral bias, we show that whereas progressive damage is pervasive in vulnerable networks, it is less severe in the general epilepsy population than previously thought based on tertiary cohort data. These findings provide a more optimistic yet realistic basis for advising people with epilepsy. The association among ongoing seizures, early disease stages, and rapid atrophy supports the notion of a critical window for early, aggressive seizure control and, when available, antiepileptogenic therapy, to prevent irreversible structural damage and preserve brain health.

## Author Contributions

R.L., A.E., L.L., M.K., and J.D. contributed to the conception and design of the study; R.L., A.E., M.G., and R.T. contributed to the acquisition and analysis of data; M.G. contributed to drafting the text and preparing the figures.

## Potential Conflicts of Interest

M.G. received speaker/advisory board fees from Advisis, Angelini, Bial, Eisai, Neuraxpharm, and UCB. R.L. received speaker fees from Angelini, Eisai, and UCB.

## Data Availability

The data that support the findings of this study are available on request from the corresponding author. The data are not publicly available due to privacy or ethical restrictions.
